# Dual topology of co-chaperones at the membrane of the endoplasmic reticulum

**DOI:** 10.1038/s41420-021-00594-x

**Published:** 2021-08-05

**Authors:** Lea Daverkausen-Fischer, Felicitas Pröls

**Affiliations:** University of Cologne, Faculty of Medicine, Institute of Anatomy II, 50931 Cologne, Germany

**Keywords:** Chaperones, Stress signalling

## Abstract

Dual topologies of proteins at the ER membrane are known for a variety of proteins allowing the same protein to exert different functions according to the topology adopted. A dual topology of the co-chaperone ERdj4, which resides in the endoplasmic reticulum (ER), was proposed recently, a thesis that we found to align all published data and existing controversies into one whole picture. The aim of this review is to reassess all primary data available in the literature on ER-resident Hsp40 co-chaperones with respect to their topology. After careful and critical analyses of all experimental data published so far, we identified, next to ERdj4, two other co-chaperones, ERdj3 and ERdj6, that also display features of a dual topology at the ER membrane. We assume that during cellular stress subpools of some ER-resident J protein can alter their topology so that these proteins can exert different functions in order to adapt to cellular stress.

## Facts

- Hsp40 co-chaperones stimulate the ATPase activity of Hsp70 chaperones via their highly conserved J-domain to enable folding or degradation of target proteins
- Hsp40 co-chaperones are largely involved in controlling as well as in restoring cellular homeostasis in stressed cells
- Seven Hsp40 co-chaperones (ERdj1–ERdj7) are known to reside in the endoplasmic reticulum and were analysed with respect to their function, their subcellular localization, and topology
- Published data indicate that three of them—ERdj3, ERdj4, and ERdj6—reside in different subcellular pools.

## Open Questions

- Further studies need to systematically address the topology of ERdj3, ERdj4, and ERdj6 in cells subjected to diverse stressors such as genotoxic stress, ER stress, nutritional deprivation, heat shock, or oxidative stress.
- The functions of these co-chaperones with respect to their topologies need to be carefully re-addressed under those stress conditions.
- The topology-dependent function of the co-chaperones needs to be analysed in diverse diseases such as neurodegenerative diseases, tumor growth and spreading, infection, inflammation, and diabetes.

The endoplasmic reticulum (ER) is the major cellular site for maintaining protein homeostasis of membrane and secretory proteins under physiological as well as stress conditions. One third of all proteins synthesized within the cell are translocated into the ER for posttranslational modification, folding, and maturation. Terminally misfolded proteins destined for proteasomal degradation are retranslocated into the cytosol while properly folded proteins can be further processed to their final destination. Within the ER, the Hsp70 chaperone BiP is crucial for folding, translocation, and degradation of proteins. In its ATP bound state BiP has a low affinity for substrate proteins, while substrate affinity is high in its ADP bound state. Binding to BiP and stimulation of its ATPase activity is mediated by the J domain of ER-resident co-chaperones, accordingly named J-proteins. Until today seven J-domain proteins, named ERdj1-ERdj7, have been identified as BiP interaction partners within the ER. Recently, data accumulate that suggest a dual topology of the ERdj4 protein with one pool being localized in the ER either being membrane-associated [1, 2] or free-floating [3] and a second ERdj4 protein pool being an integral membrane protein anchored in the ER membrane by its signal sequence and facing the cytosol [2]. In order to analyse all available experimental data with respect to the topology of ERdj4 and to elucidate whether experimental data of other J-proteins might also point to a dual topology, we reinvestigated the topology of all ER-resident J proteins characterized so far. Having carefully analysed all primary data available in the literature we suggest that in addition to ERdj4, ERdj3 and ERdj6 also seem to display a dual membrane topology under ER stress conditions.

## Mechanisms to achieve a dual topology

How can two different protein pools be generated? A possible explanation could be a dual way of signal sequence insertion into the translocon. Accordingly, the luminal protein pool is generated by the insertion of the signal sequence in the translocon in a loop formation. Inversely, a head-on formation of the signal sequence is required to generate a type I integral membrane protein pool with the C-terminus facing the cytosol [2, 4]. Whether a signal sequence inserts in a loop- or head-on way is proposed to be largely controlled by the signal sequence flanking region. Besides this, a dual topology can also be achieved by either inefficient cleavage of the signal peptide, as it is shown for EDEM1 [5], by the presence or absence of axillary proteins as shown for ERdj3 [6, 7], or by posttranslational translocation of the N-terminus, as shown for hepatitis B large envelope protein [8].

In order to assess the topology and subcellular localization of proteins, different approaches were applied. First of all, computer-based analyses give an initial hint to predict cleavage probabilities of the signal sequence and subcellular localization (Table 1). Experimental approaches include e.g., pelleting of the microsomal fraction of cells to discriminate between cytosolic and microsomal localization of the proteins. Proteinase K digestion in combination with detergents indicates whether the examined protein is located in the lumen of the endoplasmic reticulum (ER), whether it is transmembrane or an ER membrane-associated protein. To discriminate between membrane-associated and integral ER membrane proteins cell lysates can also be subjected to carbonate extraction analysis, which solubilizes associated proteins but keeps integral membrane proteins in the pellet fraction.

**Table 1:** Computational analyses of the seven human ERdJ proteins.

Length, size	Signal peptide probability	Cleavage probability	J-domain	TM domain	Other domains
Name: ERdj1, synonymous: ER1p, MTj1, HTJ126					
554 aa 63.8 kDa	79%	aa 47/48 97.4%	aa 65–126	N-terminus inside aa 30 … 48 i – o aa 154 …171 o –i aa 222 …242 i – o alternative model aa 30 … 48 o – i aa 149 …170 i – o	Myb DNA binding domain aa 329…374 aa 495…540
Name: ERdj2, synonymous: DNAJC23, PCLD2, PRO2507, SEC63L					
760 aa 87.8 kDa	None 0.2%		aa 104–161	N-terminus inside aa 15 … 35 o – i aa 76 … 92 i – o aa 193 …212 o – i alternative model aa 15 … 34 i – o aa 73 … 91 o – i aa 190 … 208 i - o	
Name: ERdj3, synonymous: DNAJB11, apobec-1-binding protein-2, PWP1-interacting protein 4, apobec-1 binding protein, HEDJ					
358 aa 40.5 kDa	84%	aa 22/23 53.2%	aa 25–87	N-terminus outside aa 6… 23 o – i aa 105…121 i – o alternative model aa 1… 23 i - o	
Name: ERdj4, synonymous: Mdg1, Mdj7, DnaJB9					
222 aa 25.7 kDa	80.4%	aa 23/24 66%	aa 26–87	N-terminus inside aa 2…22 i – o alternative model aa 2 … 22 o - i	U4/U6.U5 small nuclear ribonucleoproteins: aa 170–198
Name: ERdj5, synonymous: DnajC10, JPDI					
793 aa 91.1 kDa	None 3.3%		aa 35–97	N-terminus inside aa 17… 37 i – o aa 245 … 264 o –I alternative model aa 16 … 32 o – i aa 245 … 264 i – o	Thioredoxin domains 131 … 229 466 … 558 … 641 674 … 762
Name: ERdj6, synonymous: DnajC3, p58IPK					
504 aa 57.6 kDa	62.3%	aa 31/32 32%	aa 394–459	N-terminus outside aa 3 … 26 o – i alternative model aa 7 … 26 i - o	Multiple tetratricopeptide repeats (TPRs)
Name: ERdj7					
295 aa 34.7 kDa	99.7%	aa 22/23 49.7%	aa 29–90	N-terminus outside aa 1 … 17 o – i aa 119 … 137 i – o aa 202 … 223 o – i alternative model aa 2 … 19 i – o aa 119 … 135 o – i aa 208 … 227 i – o	

Programs used for theoretical analyses: Calculated mass: https://web.expasy.org/cgi-bin/compute_pi/pi_tool; Potential motifs and transmembrane structure https://www.genome.jp/tools/motif/.

Potential signal sequences and their cleavage probability http://www.cbs.dtu.dk/services/SignalP/.

When reassessing experimental data in the literature, we did not find any ambiguous data for ERdj1, ERdj2, ERdj5, or ERdj7 with respect to their topology.

**Fig. 1:**
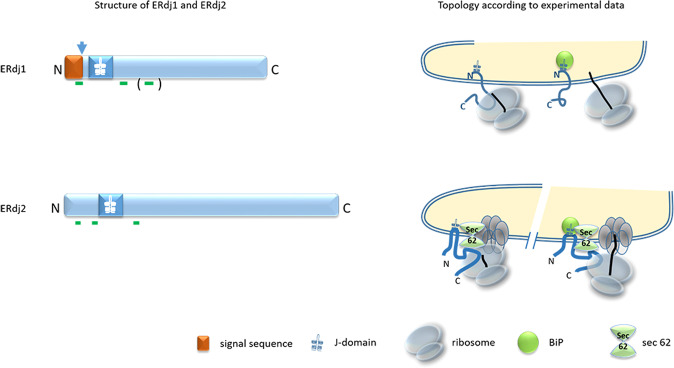
Structure and topology of ERdj1 and ERdj2. Upper line: Computational analyses of the structure of ERdj1 predicts a cleavable signal peptide and three transmembrane domains (green lines). Green lines in brackets indicate that the existence of the transmembrane domain was not confirmed experimentally. The probability of signal sequence cleavage (arrow) of ERdj1 is calculated to be 97%. Experimental data show that the first predicted transmembrane domain, which is located within the signal sequence, is cleaved upon translocation of ERdj1 into the ER. ERdj1 is anchored within the membrane of the endoplasmic reticulum by the second predicted transmembrane domain while the third predicted transmembrane domain is not used in vivo resulting in a cytosolically localized C-terminus, which interacts with the ribosome to inhibit the initiation of translation [9]. When the J-domain binds to BiP this inhibition is released and cotranslational translocation of specific proteins proceeds [9, 12, 14]. Lower line: Computational analyses of ERdj2 predicts three transmembrane regions, the orientation is likewise possible with the C-terminus either intraluminal or cytosolic. No signal sequence is identified by computational programs. Experimental data confirm two of the predicted transmembrane domains resulting in a topology with the N- and C-terminus facing the cytosol and the J-domain being located in the lumen of the ER. The C-terminus of ERdj2 interacts with Sec62 and inhibits the translation of target proteins [18]. The J-domain of ERdj2 can bind to BiP in the presence of ATP and stimulate its ATPase activity [17] releasing the translational block at the ribosomal tunnel exit.

For ERdj1 computational analyses points to three transmembrane domains and a putative signal sequence, the probability of its cleavage being high (97.4%) (Table 1). Accordingly, experimental data show that the signal peptide, as well as the first transmembrane segment, are cleaved from ERdj1 upon integration into the ER [9]. Furthermore, the third, putative transmembrane domain is not used resulting in a single transmembrane protein separating its luminal J-domain from its cytosolic C-terminus [9] (Fig. 1). ERdj1 can translocate into the ER in a Sec63 (also named ERdj2) independent manner pointing to a rather strong signal sequence of ERdj1 that can efficiently insert into the Sec61 translocon and promote the opening of the lateral gate [6, 10, 11].

The cytosolic domain of ERdj1 was shown to associate with ribosomes [9]. Experiments with different derivates of ERdj1 identified aa173–aa194 within the cytosolic domain to be responsible for ribosome binding [12]. In vitro translation assays in the presence of ribosomal subunits or ribosomal RNA as well as electron microscopy revealed that ERdj1 binds to the 60 S subunit of eukaryotic ribosomes near the tunnel exit where it can also interact with the emerging protein [12, 13] and blocks its translation. This inhibitory function requires the amino acid sequence stretch (RKKRERKKK) localized at the N-terminus of the cytosolic domain [9]. Release of translational inhibition is achieved by binding of BiP to ERdj1 [9, 12, 14]. According to the data, we hypothesize that translational inhibition through ERdj1 in the absence of its binding to BiP could present a mechanism by which a balance of chaperone to protein levels is ensured within the ER lumen. In the presence of many unfolded proteins, BiP is mainly bound to client proteins allowing ERdj1 to arrest translation at the ribosome and thereby lowering the protein burden of the ER. When client proteins have successfully been folded or degraded, BiP is available to bind to ERdj1 thereby releasing translational arrest.

For ERdj2 computational analyses gives three putative transmembrane domains allowing two topologies, one with the J-domain facing the cytosol, the other with the J-domain facing the ER lumen, and the C-terminus facing the cytosol (Table 1). This latter conformation is the one that is clearly depicted by experimental data [15–17] (Fig. 1). Yet it is still unclear whether the first predicted transmembrane domain is in fact functional, i.e., whether ERdj2 has two or three membrane-spanning regions. Experiments with proteinase K treatment and subsequent detection of ERdj2 with antibodies directed against the N-terminus, the C-terminus, or the J domain showed that neither the C- nor the N-terminus could be detected after proteinase K treatment. Only the antibody against the J-domain was able to detect a band, ~15 kDa in size. This size would fit in well with the predicted size of the ERdj2 J-domain plus the two neighboring TM domains (as shown in Fig. 1). This experimental set suggests that ERdj2 exhibits a U-shaped conformation with the C- and the N-terminus facing the cytosol and the J-domain being located within the ER-lumen. According to this model, only two of the three potential transmembrane domains do span the ER membrane, one being located between amino acid residues 93–109 and the other between amino acid residues 221–239 [15]. However, all subsequently published papers show an S-shaped orientation of ERdj2 with three transmembrane regions and the N-terminus facing the ER lumen and the C-terminus facing the cytosol [18–22]. During our extensive literature search, we did not find any experiments that support this suggested S-shaped orientation of ERdj2 in the mammalian ER membrane.

ERdj2, also known as Sec63, associates with the translocon proteins Sec61α, Sec61β, Sec61γ, and the translocon-associated protein Sec62 [16, 17]. This interaction is achieved by the C-terminal residues 734–760 of ERdj2 and the N-terminal residues 11–155 of human Sec62 as has been shown by pulldown assays and surface plasmon resonance spectroscopy [18].

Several studies examined the effect of ERdj2 on the co- and posttranslational transport of proteins across the ER membrane [23–25]. In HeLa and NIH/3T3 cells, downregulation of ERdj2 using siRNA as well as knockdown of ERdj2 in murine kidney cells did reduce translocation of a subset of proteins that are cotranslationally translocated across the ER membrane such as ERdj3, an invariant chain of the human class II major histocompatibility complex (IVC), aquaporin 2 (AQP2), and prion protein (PrP). Yet, ERdj2 downregulation did not reduce the translocation efficiency of all cotranslationally translocated proteins examined [6]. This suggests that there must be additional factors that control whether or not a cotranslationally translocated protein is dependent on ERdj2 during translocation as e.g., the signal sequence [6], the length of the protein [25], or an internal region within the mature protein [24]. As was shown for ERdj1, ERdj2 has the ability to control the protein load within the ER.

**Fig. 2:**
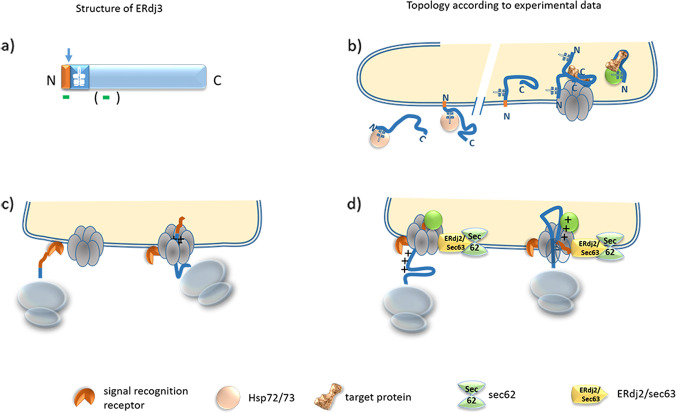
Structure and topology of ERdj3. **a** ERdj3 has a signal sequence, which has a cleavage probability of 53%. The first putative transmembrane domain is located within the signal sequence. Accordingly, cleavage of the signal sequence would give rise to a free-floating protein while the uncleaved sequence could result in its anchorage within the membrane. The second predicted transmembrane domain seems not to be functional (−). **b** Experimental data point to the existence of a cleaved and uncleaved, membrane-anchored ERdj3 protein pool, the membrane-anchored protein might be inserted into the membrane as a type I as well as a type II protein, i.e., facing the cytosol as well as the lumen of the ER. **c**, **d** The schematic drawing gives an experimental data-based model of how the prevailing (cytosolic or luminal) orientation might be regulated. **c** The head-on insertion in the absence of axillary proteins gives rise to the cytosolic orientation. ERdj3 can insert in the ER membrane but due to the weak signal sequence, it cannot flip. **d** The protein is partially translated in the cytosol. Sec62 and Sec63/ERdj2 bind to the translocon resulting in the flipping of the signal sequence and its translocation into the lumen of the ER. Symbols, which are not explained, are the same as used in Fig. 1.

ERdj3 has 358 amino acids and its predicted size is 40.5 kDa. The existence of a signal sequence is predicted with a probability of 84%, its cleavage is predicted to occur with a 53% probability (Table 1). The computational analysis predicts a signal peptide and two transmembrane domains, the first transmembrane domain being located within the signal sequence. Both transmembrane domains flank the J-domain (Fig. 2). Cleavage of the signal sequence was confirmed by experiments comparing the length of in vitro translated ERdj3 with the one of endogenous ERdj3 immunoprecipitated from Ramous cells [26]. Various experiments have shown that ERdj3 assembles as a multimer in cells [27–29]. Whereas it was first believed that ERdj3 forms dimers through its C-terminal domain [27], gel filtration experiments, and analytical ultracentrifugation, as well as electron microscopy, showed that ERdj3 assembles as a diamond-shaped tetramer (dimer of two dimers) in a medium secreted from HEK293 cells [28].

Immunostaining experiments confirmed ER-localization of ERdj3 in various cell lines [30–32]. Ambiguous data are published with respect to the topology of ERdj3. Due to its resistance to proteinase K and its glycosylation, the major pool of ERdj3 seems to be localized in the ER lumen [26, 30, 33]. Interestingly, within the ER, the majority of ERdj3 is associated with other proteins forming large protein complexes and only a minor fraction of ERdj3 is present as a free pool [34]. This finding is based on experimental results in which ERdj3 displays very low mobility [26, 30, 32] probably due to its binding to the Sec61 translocon [32], where ERdj3 promotes the degradation of target proteins via the ERAD pathway [35].

Besides this luminal pool, a minor protein pool seems to reside within the membrane. Carbonate extraction experiments showed that a fraction of ERdj3 resided in the pellet fraction similar to the results obtained for ERdj4 [36]. Thus, the results from carbonate extraction experiments point to a second, minor, integral membrane pool of ERdj3. It remains to be analysed whether this pool increases in stressed cells.

Taking into account that the presence of ERdj2, Sec62, and BiP are essential for functional translocation of ERdj3 into the ER [6, 7], an interesting model has been proposed recently according to which the insertion of a protein’s signal peptide into the translocon in a loop-formation depends on accessory proteins [10]. Most interestingly, an insertion of ERdj3 into the Sec61 translocon in a head-on formation also occurs in the absence of these accessory proteins [7] (Fig. 2c). The accessory proteins are suggested to mediate the flipping of the signal peptide from a head-on insertion to a loop-formation [10] (Fig. 2d). Thus, we hypothesize that in the absence of accessory proteins, ERdj3 signal peptide is inserted into the translocon in a head-on manner, whereas in the presence of accessory proteins it is inserted in a loop-formation thereby generating different topologies of the ERdj3. Accordingly, it would be possible that under ER stress conditions or upon inefficient translocation of ERdj3, due to unavailability of accessory proteins, ERdj3 would insert in a head-on manner in the translocon. Consequently, there would be two pools of the ERdj3 co-chaperone. The presence of two positively charged amino acids adjacent to the signal sequence of ERdj3 would fit well with the hypothesis that ERdj3 indeed favors a head-on insertion into the translocon. It would be interesting to see what happens to non-translocated ERdj3 in the cell, a question that has been addressed recently. According to these data, the majority of ERdj3 that is integrated into the ER membrane in a type I membrane topology (in the absence of BiP) is rapidly degraded via the proteasome [7]. Yet, the cleaved protein pool remained high and even increased in tunicamycin stressed cells [7]. The cleaved portion is interpreted as the protein pool residing in the ER lumen. But the cleaved pool could also—at least in part—be derived from the type I membrane fraction releasing the C-terminal domain into the cytosol by protease cleavage. This thesis would be in line with reports that allocated ERdj3 to the cellular cytoplasm and even discussed it to function as a transcription factor in the nucleus. In fact, experimental data showed that ERdj3 is able to bind the cytosolic/nuclear Hsp70 chaperones Hsp72 and Hsp73 and an ERdj3 variant, attached to the cytosolic side of the ER membrane, was shown to stimulate the ATPase activity of cytosolic localized yeast Hsp70 chaperone Ssa1 when expressed in yeast [37, 38]. When thinking of ERdj3 as a transcription factor, it must be cleaved at the membrane to be enabled to travel to the nucleus. Experiments have to be performed to clearly distinguish between cytosolic, nuclear and luminal protein pools. It would be interesting to see whether ER stress alters the topology of ERdj3 as has been suggested for ERdj6 (see below).

ERdj4 contains an N-terminal signal sequence (aa 1–23), which is followed by a J domain (aa 24–93) (Table 1). Immunocytochemical analyses of transfected cos7 and HEK293T cells overexpressing ERdj4 localized ERdj4 to the ER [3, 8, 36, 39]. However, ERdj4 could not be detected in dog pancreatic microsomes under control conditions [20]. The reason for this is very likely based on the fact that under physiological conditions ERdj4 levels are very low [40]. ERdj4 levels were shown to drastically increase in ER-stressed cells [3, 39–41]. High levels of ERdj4 have been shown to promote cell survival in response to ER stress [39]. Also during B cell development, ERdj4 seems to protect precursor B cells from apoptosis [42] and overexpression of ERdj4 could improve engraftment of transplanted human stem cells possibly by preventing CHOP and GADD34 induction [43]. This prosurvival effect of ERdj4 is very likely based on its “surveillance” ability since it has been shown to target aggregation-prone mutated proteins to transfer them to the ER-associated degradation (ERAD) pathway [35, 44–46]. According to this function, the major pool of ERdj4 is located in the ER. Conflicting data exist with respect to the topology of ERdj4, i.e., whether it is a transmembrane or luminal protein. As a transmembrane protein, ERdj4 should be inserted into the membrane by its signal sequence, cleavage of the signal sequence would give rise to a luminal protein pool. The computational analysis predicts cleavage of the signal peptide to occur with a probability of 66% (Table 1). The data analysing signal cleavage of the ERdj4 protein are not clear cut. Data based on gel electrophoresis experiments in the presence or absence of microsomes indicate that the signal sequence is not cleaved [3, 39]. In addition, digitonin treatment showed ERdj4 to behave more like an integral membrane protein than a soluble ER luminal protein [3]. Recent data revealed that it is only a minor pool of ERdj4 that exists as an integral membrane protein [1, 2]. The major part of ERdj4 could be solubilized from ER membranes by sodium carbonate extraction [36]. In addition, computational analysis as well as Erdman digestion and high-resolution gel electrophoresis showed cleavage of the signal sequence [36] and photobleaching experiments, further, demonstrated that ERdj4 is mobile throughout the whole ER and that mobility of ERdj4 increases when it is bound to BiP [36].

**Fig. 3:**
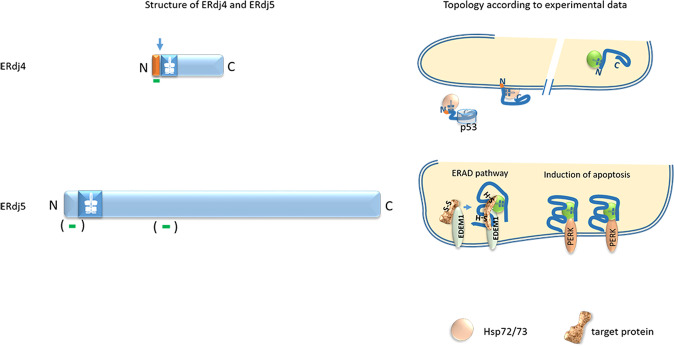
Structure and topology of ERdj4 and ERdj5. Upper line: ERdj4 is a small protein with a predicted size of 25.7 kDa, a signal peptide (80% probability), the cleavage of which is calculated with a probability of 70%. As for ERdj3, the signal sequence contains the hydrophobic region addressed as the transmembrane domain. Cleavage of the signal sequence (arrow) accordingly results in a “free-floating” protein, either in the lumen of the ER or in the cytosol. The major pool of ERdj4 protein is cleaved and located in the luminal compartment of the ER. A minor pool exists as an uncleaved transmembrane protein. Increasing evidence points to a cytosolic orientation or even a cytosolic pool that can shuttle to the nucleus. Lower line: Computational analysis of ERdj5 predicts no signal sequence but two transmembrane domains. Experimental data do not confirm the existence of transmembrane domains. ERdj5 seems to be entirely located within the ER lumen, where it interacts with EDEM1 to reduce EDEM1-recruited substrates to enable their degradation via ERAD [61]. Due to its complexing with BiP and PERK, ERdj5 inhibits autophosphorylation of PERK and subsequent phosphorylation of eIF2α inducing apoptosis as shown in colon cancer cells [63]. Symbols, which are not explained are the same as in Fig. 1.

All in all, the data could as well point to the existence of two different pools of ERdj4, a model proposed recently [2] (Fig. 3). Evidence for these two different pools came from proteinase K experiments of whole-cell lysates of Hepa 1–6 cells stably expressing GFP tagged ERdj4. The experiments showed that only 75% of ERdj4 were protected from proteinase K treatment. Thus, the authors proposed that 25% of ERdj4 should be located cytosolically with the signal peptide anchoring the protein within the ER membrane [2]. This model is supported by a DeepLoc computational analysis conducted by us that predicts around 70% of ERdj4 to be present as a luminal protein and 30% of the protein to be present as an integral ER membrane protein.

Cytosolic localization of ERdj4 was already predicted by PSORT computational analysis [47]. In cells stressed by genotoxic agents, ERdj4 forms protein–protein complexes with p53 which can be immunoprecipitated in cytosolic and nuclear fractions and prevent p53-induced apoptosis [48]. Furthermore, immunohistochemical stainings of lymphocytes and granulocytes from dermal chronic wound tissue showed ERdj4 positive stainings of the nuclei [49, 50]. In heat-shocked cells, immunocytochemical experiments showed that ERdj4 translocates from the ER to the nuclear nucleoli, a translocation that could be reversed upon recovery from heat shock [40, 41]. When assessing ERdj4 expression in fibrillary glomerulonephritis pathology, IHC was performed on various healthy human tissue types many of which showed granular cytoplasmic staining for ERdj4 supporting a cytoplasmic localization of ERdj4 [51].

Murine ERdj5, also known as JPDI, was first discovered in 2002 by EST database search [52]. Computational analyses predict no signal sequence and two transmembrane domains, localizing the J-domain either cytosolically or luminally within the ER (Table 1). Yet, experimental data locate the glycosylated protein ERdj5 exclusively in the lumen of the ER [52]. Although no signal sequence is predicted by computational analyses, signal sequence cleavage was postulated according to the mass detected by gel electrophoresis [52]. Localization within the ER lumen was further validated by its complete resistance to proteinase K treatment in microsomes isolated from HeLa cells [52] and by its glycosylation as evidenced by EndoH treatment of lysed HeLa cells [52] (Fig. 3).

Confocal immunofluorescence microscopy localized various tagged constructs of ERdj5 to the ER [52–56], two of the studies used PDI as an ER marker [52, 56], other studies used pDsRed2-ER or BiP as ER markers which altogether point to the unique picture of ERdj5 being localized in the lumen of the ER. Furthermore, ERdj5 contains a C-terminal KDEL sequence, which is thought to function as ER retention signal [52].

The ERdj5 protein possesses a hydrophobic N-terminal sequence followed by a J-domain of 66 amino acids and six tandem thioredoxin domains [57], a unique feature among the J-domain proteins. The crystal structure of full-length ERdj5 revealed that the thioredoxin domains are arranged in a N- and C-terminal cluster. The C-terminal cluster was shown to interact with EDEM1 to reduce the EDEM1-recruited substrates [57] and to accelerate ERAD of a variety of target proteins [58, 59]. Both the reductase activity of ERdj5, which is conferred by its CXXC motifs, and the association of ERdj5 with the molecular chaperone BiP [55, 60, 61] are necessary to prevent multimer formation of misfolded proteins and to promote efficient ERAD [61]. Besides its function in the ERAD pathway, ERdj5 is also required for efficient folding of specific target proteins as was shown for maturation of the LDL receptor [56]. Furthermore, ERdj5 inhibits phosphorylation of eIF2α (via PERK) thereby inducing cellular apoptosis as has been observed in ERdj5 overexpressing cells and in colon cancer cells [62–64]. Since all data point to the exclusive luminal localization of ERdj5, inhibition of PERK phosphorylation could be achieved by ERdj5-mediated stabilization of the BiP/PERK complex thereby silencing PERK activity and subsequent phosphorylation of eIF2α.

ERdj6: Computational analysis predicts ERdj6 to have a signal sequence of 31 amino acids in length. Its cleavage is only predicted with a probability of 32% (Table 1). The experimental data destined to elucidate the subcellular localization of ERdj6 and its topology at the ER membrane are not clear cut. An import of ERdj6 into the ER with subsequent cleavage of the signal peptide has been demonstrated [65, 66]. Furthermore, ERdj6 was shown to interact with BiP and unfolded proteins in the ER lumen [65, 66]. Also, treatment of isolated microsomes or whole-cell lysates with proteinase K revealed protection of the ERdj6 protein pool in control and ER stressed cells arguing for a luminal orientation of ERdj6 [65]. As a luminal protein, ERdj6 facilitates maturation and post-ER transport of proteins [65, 67, 68]. In vitro translocation experiments showed that not all of the protein is protected against proteinase K treatment pointing to the existence of a minor cytosolic pool [65]. In these experiments, complete resistance to proteinase K treatment and accordingly a complete translocation into the ER was achieved when exchanging the ERdj6 signal sequence for a strong signal sequence [65]. The weak endogenous ERdj6 signal sequence further argues for an increased likelihood of a dual location of ERdj6 protein. Furthermore, cytosolic Hsc70 has been shown to be recruited to ERdj6 [69] and by direct binding to the cytosolic kinase domains, ERdj6 inhibits PKR, PERK, and GCN2 kinase activities [70–75]. The cytosolic pool is supposed to be very small but very efficient in controlling phosphorylation of eIF2a, thereby promoting general protein translation [72], a mechanism extremely important for secretory active cells such as immunoglobulin-secreting plasma cells.

**Fig. 4:**
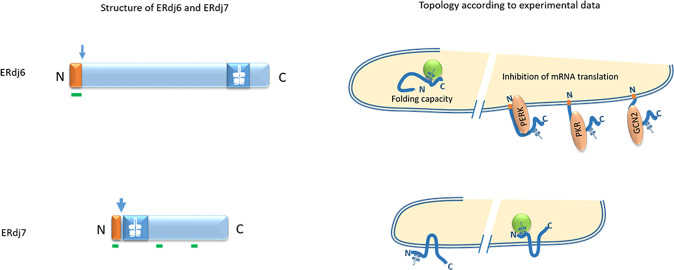
Structure and topology of ERdj6 and ERdj7. Upper line: Computational analysis of ERdj6 predicts the presence of a signal sequence with a probability of 62%, the probability of its cleavage to be 32% (arrow), and a single transmembrane domain, which is located within the signal sequence. The experimental data point to cleavage of the signal sequence giving rise to a major, luminal free-floating ERdj6 protein pool, which catalyze ATPase activity of BiP to facilitate folding of luminal proteins [65, 72]. A minor Erdj6 pool remains uncleaved and inserts in a head-on formation as a type I transmembrane protein. In this topology, ERdj6 can physically interact and inhibit the kinase domain of PKR, PERK, and GCN2 thereby inhibiting phosphorylation of eIF2α and promoting protein synthesis [65, 72]. Lower line: Computational analysis of ERdj7 predicts the existence of a signal sequence with a cleavage probability of 50% (arrow). Three putative transmembrane domains are predicted, the first one is located within the signal sequence. No experimental data are available that investigated the orientation of ERdj7 at the ER membrane. Provided that the signal sequence is cleaved upon translocation and the existence of two membrane-spanning regions the topology could be as suggested in the diagram with the J-domain being either located luminal or cytosolic. Symbols used are described in Fig. 1.

All in all these experimental data points to a dual topology of ERdj6: As a major luminal, free-floating pool, ERdj6 interacts with BiP and primarily controls maturation and secretion of proteins that transit the ER thereby reducing the protein load within the ER, a function relevant under normal conditions and during early stress conditions [65]. A minor but functionally relevant pool resides at the ER membrane with the C-terminus facing the cytosol (Fig. 4). Such a dual localization is rather likely to be achieved by proteins that have such a weak signal peptide as ERdj6. The dual topology at the ER membrane corresponds very well with the dual functions suggested by Yan et al. already in 2002 [76]: “a J-domain dependent function required for protein folding within the ER and a J-domain independent function to block kinase activities of PKR and PERK and subsequently the phosphorylation of eIF2α to release the protein translational block in the cytosol”. This late stress response is required to restore the cellular metabolism in cells that either adapted to the altered microenvironment, originally sensed as stress, but also in cells that can no longer cope with the stress condition and initiate apoptosis.

Very little information is available with respect to the topology of ERdj7, which was first cloned in mammalian cells in 2012 [77]. By overexpressing an ERdj7-GFP fusion construct in HeLa cells ERdj7 was described to be located in the cytoplasm. When critically analysing the documentation of the fluorescent pictures, especially in comparison to the control GFP-transfected cells, it seems that the overexpressed fusion construct forms aggregates and the fluorescence emitted from non-aggregated protein is not uniformly distributed in the cytoplasm but forms a reticular pattern, which might very well be due to localization in the ER compartment [77] (Fig. 4). Interestingly, expression of ERdj7 is low in hepatocellular carcinoma cells and overexpression of ERdj7 has been shown to promote apoptosis [77]. Low pH and hypoxic conditions prevail in solid tumors, conditions shown to downregulate ERdj7 supporting the thesis that low ERdj7 levels might enable tumor growth and that overexpression of ERdj7 might inhibit tumor growth by inducing apoptosis. Nothing is yet known about the underlying molecular mechanisms.

## Concluding remarks

The topology of the J-protein is not just an intellectual problem but has, due to compartment-specific interaction partners, functional implications [5, 8]. Having reviewed all available data on topology and subcellular localization of the mammalian ER co-chaperones we reveal evidence that—according to their signal sequence characteristics, the ambiguous data on their subcellular localization as well as their topology—ERdj3, ERdj4, and ERdj6, have the potential for a dual membrane topology under ER stress conditions (Table 2). In the case of ERdj6, it was shown that ER stress induced by thapsigargin treatment can affect the distribution of topologically different protein pools [65]. ERdj4 translocates to the nucleus in pathological conditions and in heat-stressed cells [40, 41, 50], a protein pool that very likely stems from the cytosolic protein fraction. Therefore, it would be interesting to assess the topology of ERdj3 under conditions of increased cellular stress. Interestingly, the orientation (head-on or loop-formation) of the signal peptide at the ER membrane is directed by the presence or absence of BiP and ERdj2 [6, 7, 10]. Since the signal sequence of ERdj3 has no charged residues but is neighbored by two positively charged amino acids, in the absence of ERdj2 and BiP, ERdj3 indeed favors a head-on insertion into the translocon.

**Table 2:** Topology and function of ERdj proteins.

	Topology	Function
ERdj1	Integral membrane protein with a luminal J-domain and cytosolic C-terminus	Binding to the ribosome (60 S subunit) near the tunnel exit In the absence of BiP: translational block In the presence of BiP: translational release
ERdj2	Integral membrane protein with the luminal J-domain and a cytosolic C-domain	At the cytosolic site: association with the translocon proteins Sec61 α, β, γ, and Sec62 Transport of proteins across the ER membrane to control the protein load in the ER
ERdj3	Within the ER lumen: Major pool: part of large protein complexes Minor pool: as free protein pool --------------------------------------------- Integral membrane protein: minor pool In the absence of accessory proteins: head-on insertion (cytosolic orientation) In the presence of accessory proteins: loop insertion (luminal orientation)	Interaction with the Sec61 translocon Promoting protein degradation via ERAD --------------------------------------------- Putative transcription factor in the nucleus
ERdj4	70–75% of the protein: soluble in the ER lumen --------------------------------------------- 25–30% of the protein: integral membrane protein facing the cytosol	Targeting of proteins to the ERAD pathway Protein folding Induction of apoptosis ---------------------------------------------
ERdj5	Luminal localization	Acceleration of the ERAD pathway Protein folding Apoptosis
ERdj6	Major pool: luminal localization --------------------------------------------- Minor pool: integral membrane protein facing the cytosol	Protein maturation and post-ER transport of target proteins --------------------------------------------- Inhibition of protein synthesis by - Direct binding to the kinase domains of PKR, PERK, and GCN2 kinases - Very efficient control of eIF2a phosphorylation
ERdj7	ER-localization	

As ERdj2/BiP dependency during translocation might be tightly linked to the topology of other proteins, it would be of interest to assess the ERdj2/BiP dependency of all the other ER J-proteins during their translocation into the ER. The signal sequence of ERdj4 is very similar to the one of ERdj3 in terms of the lack of charged residues as well as hydrophobicity as was assessed by Kyte&Doolittle hydrophobicity blots arguing for a similar mode of signal sequence insertion into the translocon.

All in all, we think the presence of dual topologies and different subcellular pools of J proteins has important impacts on cellular functions under different conditions. For a variety of proteins, such as EDEM1, ERdj4, the large envelope protein of the Hepatitis B virus and diacylglycerol-acyltransferase 1 (DGAT1) it was shown that when adopting different topologies, the same protein exerts different functions [2, 5, 78, 79]. Likewise, ER stress is shown to affect the topology of ERdj6 and the secretion of ERdj3 [65, 80]. In the case of ERdj6, the pro-folding function of the ER-resident protein pool could be matched to the substrate synthesis rate controlled by the cytosolic domain of ERdj6 by its binding to the PERK kinase domain thereby releasing translational arrest during the recovery phase of the unfolded protein response [65]. The switch in subcellular pools of J-proteins is a reasonable tool to adapt to the altered cellular environment in stressed cells, exert different functions, and adapt to ER stress.
